# Characterization of a reassortant H11N9 subtype avian influenza virus isolated from spot-billed duck in China

**DOI:** 10.1007/s11262-023-02009-8

**Published:** 2023-06-02

**Authors:** Bo Wang, Yanyi Huang, Bin Hu, Heng Zhang, Shuyi Han, Ziwen Yang, Qianqian Su, Hongxuan He

**Affiliations:** 1 National Research Center for Wildlife-Borne Diseases, Institute of Zoology, Chinese Academy of Sciences, No. 1-5 Beichenxilu, Chaoyang District, Beijing, 100101 People’s Republic of China; 2 College of Life Science, University of Chinese Academy of Sciences, Beijing, China

**Keywords:** H11N9, Phylogenetic analyses, Reassortment, Wild bird

## Abstract

H11N9 viruses in wild birds might have provided the NA gene of human H7N9 virus in early 2013 in China, which evolved with highly pathogenic strains in 2017 and caused severe fatalities. To investigate the prevalence and evolution of the H11N9 influenza viruses, 16,781 samples were collected and analyzed during 2016–2020. As a result, a novel strain of influenza A (H11N9) virus with several characteristics that increase virulence was isolated. This strain had reduced pathogenicity in chicken and mice and was able to replicate in mice without prior adaptation. Phylogenetic analyses showed that it was a sextuple‐reassortant virus of H11N9, H3N8, H3N6, H7N9, H9N2, and H6N8 viruses present in China, similar to the H11N9 strains in Japan and Korea during the same period. This was the H11N9 strain isolated from China most recently, which add a record to viruses in wild birds. This study identified a new H11N9 reassortant in a wild bird with key mutation contributing to virulence. Therefore, comprehensive surveillance and enhanced biosecurity precautions are particularly important for the prediction and prevention of potential pandemics resulting from reassortant viruses with continuous evolution and expanding geographic distributions.

**Supplementary Information:**

The online version contains supplementary material available at 10.1007/s11262-023-02009-8.

## Introduction

Avian influenza viruses are ribonucleic acid viruses of the family Orthomyxoviridae and possess 8 negative-sense RNA segments encoding 11 known proteins [1]. Of these, the two major surface antigens, hemagglutinin (HA) and neuraminidase (NA), form the basis of multiple serologically distinct virus subtypes. With 18 hemagglutinin (H1–H18) and 11 (N1–N11) neuraminidase subtypes, there is considerable antigenic differences among influenza viruses [2, 3]. Currently, 16 HA and 9 NA subtypes combinations exist in harmony with wild waterfowl, the major natural reservoir for all influenza A viruses, cause no overt disease, and emerge to infect domestic poultry and occasionally mammals [2, 4].

Influenza A viruses can be divided into two distinct groups of high or low pathogenicity on the basis of their pathogenicity in chickens. All highly pathogenic avian influenza viruses (HPAIVs) known to date that mutate from Low pathogenic avian influenza viruses (LPAIVs) have been restricted to subtypes H5 and H7 [5, 6]. HPAIVs infections caused by H5 and H7 subtypes in humans have been placed on the top priority list among other zoonotic avian influenza viruses (AIVs) and have raised concerns that a new influenza pandemic will occur in the future [7, 8]. H7N9 infections caused significant negative impacts on public health, the economy, and national and even global security that had resulted in 1567 human cases with 615 deaths. Human H7N9 has almost disappeared in 2018 because the effective response including management of LPMs and the vaccination strategy [9, 10]. However, H7N9 AIVs isolated in 2019 were antigenically distinct from the vaccine strain, so that the H7N9 AIV has not been eradicated from poultry in China [11].

The NA gene of human influenza A(H7N9) virus might have originated from influenza A (H2N9, H4N9, H11N9) viruses that circulated in eastern China [12–14]. Most H11N9 strains usually were found in wild birds. Some studies have described that H11N9 isolated in China can replicate in mammalian cells in vitro [15], and even in mice in vivo without prior adaption [16, 17]. Although, no H11N9 virus was isolated from human till now, serologic evidence of human past infection with influenza A/H11N9 suggested a potent risk of direct transmission of AIV to humans [18–20].

Considering that H11N9 viruses might contributed to H7N9 reassortment and have the threat to public health, active surveillance on influenza was required urgently. Recently, H11N9 viruses were detected in China (Feb 2016) [17] and South Korea (2016–2018) [19, 20]. However, the prevalence of them afterward in China was unclear. Thus, this study focused on the surveillance of H11N9 viruses during 2016–2020 to analyze their evolution and epidemic risk. As a result, we isolated one strain of the H11N9 influenza virus in Shanghai in November 2016, and analyzed the genetic origin of it, indicating that it was a local inter-subtype reassortant present in China and might be transmitted to Japan and South Korea, which prompted us to conduct further influenza surveillance in wild birds in the future.

## Methods

### Virus isolation and sequence analysis

To respond to the H7N9 outbreak, a total of 16,781 swab and fecal samples were collected from waterfowl in natural reserves in Shanghai and Jiangxi during 2016–2020. For virus isolation, 9 10-day-old specific-pathogen-free (SPF) embryonated chicken eggs were inoculated with the sample supernatants. Viral RNA was extracted from 200 ml of allantoic fluid and subjected to reverse transcriptase polymerase chain reaction (RT-PCR). First, reverse transcription was performed with the primer Uni12 and GoScript™ Reverse Transcriptase System (Promega). This was followed by PCR in which the reverse transcription product(s) was amplified by the universal primer set MBTuni-12 and -13 to amplify the short segments for hemagglutinin (HA), nucleoprotein (NP), neuraminidase (NA), matrix (M), and nonstructural gene (NS). Segment-specific primers were used to amplify the long segments for polymerase basic protein 2 (PB2), polymerase basic protein 1 (PB1), and polymerase acidic protein (PA) (Table S1) [21, 22]. Full-genome sequences of the new isolates were annotated using the Influenza Virus Sequence Annotation Tool of the Influenza Virus Database of the National Center for Biotechnology Information (NCBI; https://www.ncbi.nlm.nih.gov/genomes/FLU/annotation), then deposited in GenBank. The sequence feature was annotated on Influenza Research Database (IRD;https://www.fludb.org/brc/sequenceFeatureDetailsReport.spg?decorator=influenza&seqFeaturesNamesId=564986.).

For phylogenetic analysis, the sequences of full genomes of the top 100 basic local alignment search tool (BLAST) hits for the new isolate were downloaded from NCBI https://www.ncbi.nlm.nih.gov/genomes/FLU/Database/nph-select.cgi?go=database) and the EpiFlu database from the Global Initiative on Sharing All Influenza Data (GISAID; https://platform.epicov.org/epi3/cfrontend).

### Bayesian maximum clade credibility phylogeny

Multiple sequence alignments were produced using MUSCLE in MEGA7. Maximum clade credibility phylogenetic trees were generated for the full-genome sequences of the top 100 BLAST hits. ModelTest-NG was used to select the best-fit model for nucleotide substitutions. We used relaxed molecular clock models (uncorrelated exponential clock models) to estimate divergence times. Markov chain Monte Carlo (MCMC) chains were run for 100–1100 million iterations according to the number of sequences. The best-fit substitution and tree models are listed in Table S2. TRACER 1.6 was used to confirm appropriate burn-in and adequate effective sample sizes (ESS > 200) for the MCMC analyses [23]. All phylogenetic trees, visualized using Figtree, are presented in Supplementary Fig. 1.

### Pathogenicity in chicken and mice

To determine pathogenicity of the influenza virus in chickens, Intravenous Pathogenicity Index (IVPI) was essentially conducted as described in OIE manual [24]. Specific-pathogen-free (SPF) white leghorn chickens were purchased from Boehringer Ingelheim, Beijing, China and raised until they were 6 weeks old. A total of 10 chickens were used for one virus isolate. The serum was sampled on 10 days post infection and tested for serum conversion by hemagglutination inhibition (HI) [25].

To determine pathogenicity of the influenza virus in mice, groups of 6 6-week-old female SPF BALB/c mice (weighing ≥ 18 g) (Boehringer Ingelheim, Beijing, China) were inoculated intranasally with 10^3^ EID50 of designated virus in a volume of 50 µl. The control group was injected with an equal volume of phosphate-buffered saline. On 3 days post inoculation (dpi), three inoculated mice were sacrificed, and we collected organs including lungs, nasal turbinate, spleen, trachea, brain, and colon for viral titrations by Real-Time Polymerase Chain Reaction (qPCR). The results were extrapolated from corresponding standard curves and expressed as EID50/g equivalents [26]. The remaining three mice and control group were monitored daily up to 14 days for weight and mortality.

## Results

### Molecular characterization of the H11N9 isolates

A novel strain of the H11N9 subtype AIV was isolated from a spot-billed duck and named A/Anas poecilorhyncha/shanghai/SH2/2016 (SH2; GenBank accession numbers MW575006–MW575013). SH2 harbors a single basic residue at the cleavage site and was classified as an LPAI virus [27] (Table 1). Six residues in the HA protein (i.e., A138, E190, L194, G225, Q226, and G228; H3 numbering) were conserved, contributing to the strain’s avian receptor-binding characteristics [28]. Multiple virulence-increasing substitutions were present in SH2, including L89V, G309D, T339K, R477G, I495V, K627E, and A676T in PB2, all of which have been shown to increase virulence and replication in mammals [29]. SH2 contained residues R57, I62, S65, and V100 in PA, which suppress host-cell protein synthesis during infection, attenuating the antiviral response [30]. The putative zinc-finger motif CCHH in helix 9 of M1 in SH2 also plays a critical role in virulence in mice [31]. Remarkably, SH2 had S42 and avian-like NS1 C-terminal PDZ domain ligand (PL) residues of ESEV in the NS1 protein, which appeared to increase virulence in mice [32–35]. Interestingly, two highly conserved NS1 residues in 99% human influenza A viruses that enhance virulence in mice, F at 103 and M at 106, are also present in SH2 [36]. These H11N9 characteristics appear to have considerable pathogenic potential for humans.

**Table 1:** Genetic characteristics of the SH2 isolate from wild birds

Gene	Sequence variation	Sequence feature	References
HA	PAIASR↓GLF	Low pathogenic cleavage sites	[27]
HA (H3 num)	A138, E190, L194, G225, Q226, G228	Conserved characteristics contribute to avian receptor-binding	[28]
PB2	K627E, N701D	Decreased virulence and replication efficiency	PubMed: 21849466; PubMed: 16140781
PB2	L89V, G309D, T339K, R477G, I495V, K627E, A676T	Increased polymerase activity	[29]
PB1	Y436H	Decreased virulence in mice	PubMed: 17553873
PA	Q57R, V62I, L65S, A100V	Increased production of viral proteins	[30]
NP	A184K	Increased replication and pathogenicity in chickens	PubMed: 19475480
M1	N30D, T215A	Increased virulence	PubMed:19117585
M1	C148, C151, H159, H162	CCHH increased virulence in mice	[31]
M2	S50C	Modest increasement in virulence in mice	PubMed: 19553312
NS1	80–84 delete	Attenuated in virus replication in vitro and in vivo (chicken and mice)	PubMed: 20854176
NS1	P42S	Increased virulence in mice	[34]
NS1	N101D	Increased virulence in mice	PubMed: 10873787
NS1	L103F, I106M	Increased virulence	[36]
NS1	E227, S228, E229, V230	ESEV increased virulence in mice	[35]; [33]

### Identity analysis

**Fig. 1:**
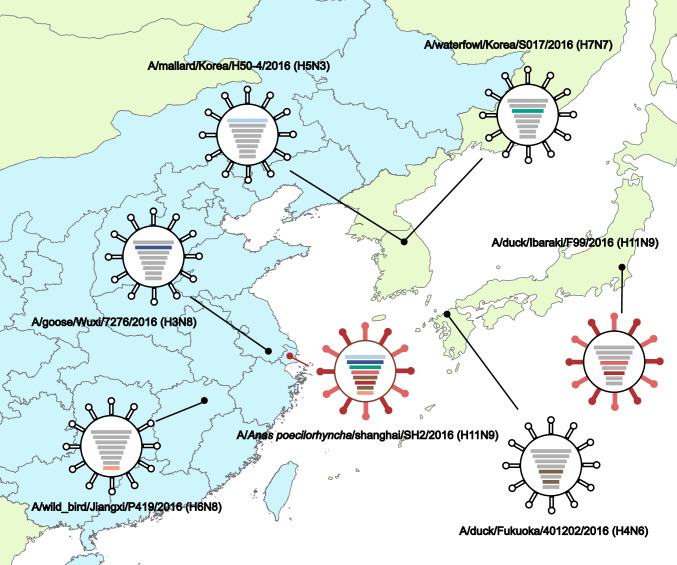
Location of viruses related to the SH2 (H11N9) virus

Next, nucleotide sequence similarity was conducted to investigate the relationships between SH2 with other influenza A viruses. We found that the HA, NA, NP, and M genes of SH2 were most closely related to viruses in Japan with 99.50%–99.94% identities, while the other SH2 inner genes (PB2, PB1, PA, and NS) were most closely related to isolates from Korea and China (Table S3). Phylogenetic analysis showed similar results (Fig. 1; Fig. S1): the surface genes HA and NA were clustered together with those of the A/duck/Ibaraki/99/2016 H11N9 strain, with 99.50% and 99.7% nucleotide identities, respectively; the internal genes NP and M were clustered with those of A/duck/Fukuoka/401202/2016 (H4N6), with identities of 99.94% and 99.80%, respectively; and the internal genes PB2 and PA were most closely related to the strains isolated in Korea. However, the PB1 and NS genes were related to the newly isolated viruses from wild waterfowl in China. The spatial locations of all virus strains, including those related to the SH2 strain, are illustrated in Fig. 1.

### Phylogenetic analysis

**Fig. 2:**
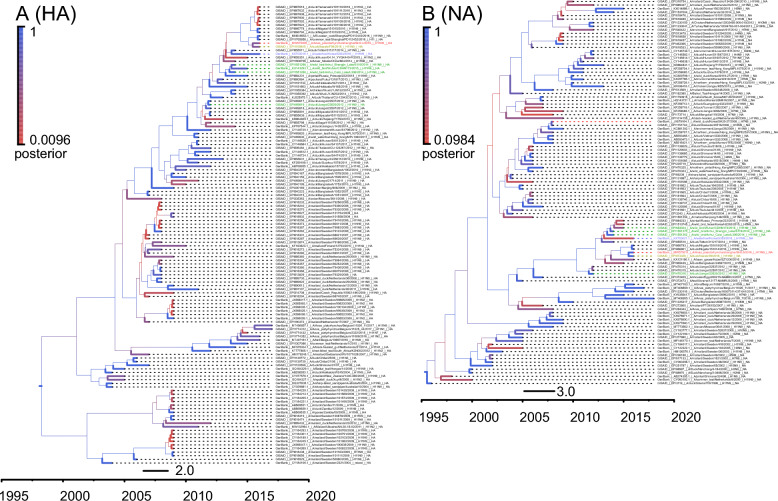
Genesis analysis of HA and NA genes. The phylogenetic trees were conducted using gene sequences of top blast hits for SH2 in the NCBI and GISAID. SH1 is marked in red, and other related viruses in China, Japan, Korea and Mongolia are marked in green, yellow, purple and blue correspondingly. All the trees were built by BEAST (v 1.8.4) and displayed using FigTree (v 1.4.2)

**Fig. 3:**
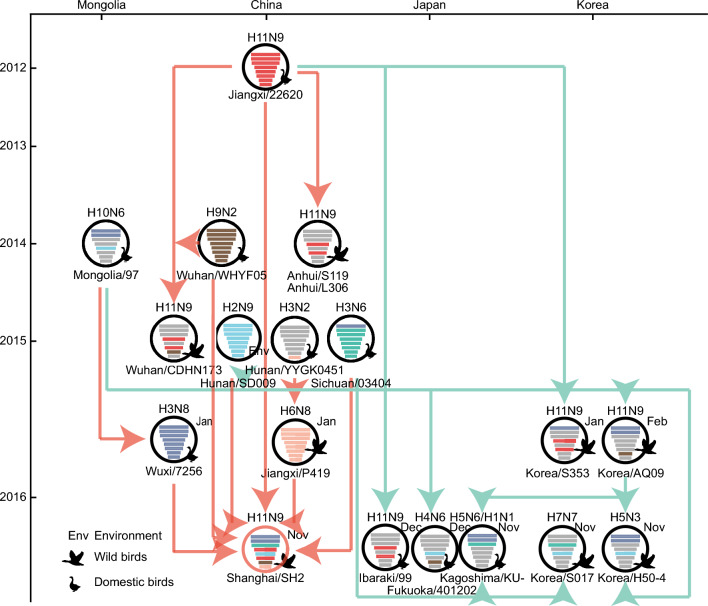
Hypothetical reassortment pathways for the genesis of SH2 virus. Virus particles are represented by colored ovals containing horizontal bars that represent the eight genome segments (top to bottom: PB2, PB1, PA, HA, NP, NA, M, and NS). Segments in descendant viruses (down) are the same color as those in their ancestor viruses (top) to illustrate reassortments. The source viruses for reassortment are adjacent to arrow tails and arrowheads point to the reassortants. The timeline on the left indicates dates of virus emergence. The top axis indicates the country of virus isolation

We then conduct phylogenetic analyses to identify genomic sources of the SH2 virus. The results showed that all eight genes originated from southern China (Fig. 2; Fig. S1-6). The HA and NA genes originated from an A/duck/Jiangxi/22620/2012 (H11N9)-like gene pool. The PB2 and PB1 genes originated from the A/goose/Wuxi/7276/2016 (H3N8)‐like gene pool, and the PA, NP, M, and NS genes originated from A/EN/Sichuan/03404/2015 (H3N6)‐like, A/environment/Hunan/SD009/2015 (H7N9)‐like, A/duck/Wuhan/WHYF05/2014 (H9N2)‐like, and A/wild bird/Jiangxi/P419/2016 (H6N8)‐like gene pool, respectively. Meanwhile, we found that the H11N9 viruses isolated during 2016–2017 in Japan and Korea were similar with SH2 and shared the same origin. Overall, SH2 is a sextuple‐reassortant virus of H11N9, H3N8, H3N6, H7N9, H9N2, and H6N8 viruses in China. This was the most recent report of the H11N9 virus in China, indicating that it circulated in this country until late 2016, and may have been transmitted to Japan and Korea (Fig. 3).

### Pathogenicity in chicken and mice

To evaluate the pathogenicity of SH2 H11N9 isolate in chicken, 6-week-old SPF chickens were injected intravenously with H11N9 influenza virus and observed for clinical signs over a period of 10 days. All inoculated chickens survived without showing any clinical signs, thus the IVPI score remained zero for studied H11N9 virus. Infection was confirmed by virus isolation on 3 and 10 dpi in lung, oropharyngeal and cloacal swabs. M gene of AIV was positive in cloacal swabs and undetectable in the lung (Table 2). HI antibody titer in inoculated chickens was measured to investigate the specific antibody response. The HI antibody against SH2 were detected at 10 dpi in two chickens. Seroconversion rate was 1/4 and HI titers were 2^3^–2^4^. Consequently, tested H11N9 virus has to be considered as low pathogenic.

**Table 2:** Analyses of virulence of H11N9 strain and antibody response in chickens

dpi	Infectivity of SH2 to chickens			IVPI	Serological conversion	
	Oropharynx (%)^a^	Cloaca (%)^b^	Lung (%)^c^		Conversion (%)^d^	HI titer range
3	0	12.5 (1/8)	0			
10	0	25 (2/8)	0	0	25 (2/8)	1:8–1:16

^a, b, c^Number shows percentage of viral positivity rate in oropharyngeal swab, cloacal swab, and lung tissue samples respectively and number in parenthesis represents number of samples in which virus was detected/number of samples examined

^d^Number shows percentage of seroconversion rate and number in parenthesis represents number of chickens in which antibody was detected/number of chickens examined

**Fig. 4:**
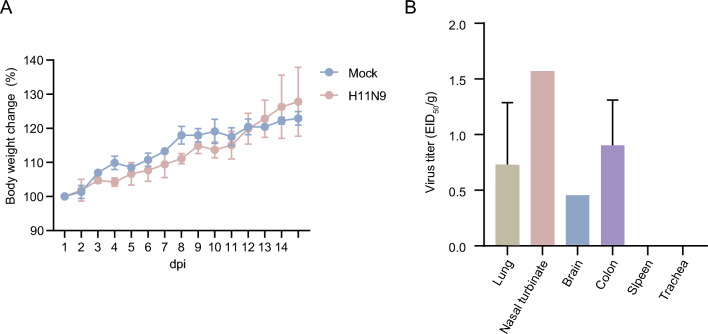
Pathogenicity of H11N9 virus in mice. Balb/c were intranasally challenged with 10^3^ EID_50_ of virus. Mice weight were observed for 14 dpi and presented as % of those of original mice (*n* = 3; A). Mean virus titers in the lungs, nasal turbinate, brain, spleen, and colon of mice (*n* = 3) were measured at 3 dpi (B)

To measure the pathogenic potential of the H11N9 virus in mammal, mice were injected intranasally with H11N9 influenza virus and recorded for weight change over a period of 14 days. In the 6-week-old mice, no death or significant weight loss were observed both in the H11N9 and mock groups (Fig. 4A). To assess for systemic virus spread, various organs were sampled for virus titration at 3 dpi from mice with 10^3^ EID50 of virus. The tested avian H11 isolates proliferated in mice nasal turbinate without pre-adaptation and with virus titer of 1.57 EID50 (Fig. 4B). Systemically, virus was detected in the lung, brain, and colon with titers ranging from 0.45 to 0.90 EID50. No virus titers were detected in the spleen and trachea.

## Discussion

The SH2 H11N9 virus isolated in Shanghai in late 2016 did not have a multiple basic amino acid sequence at the HA cleavage site (HACS), and caused no clinical signs in chickens with an IVPI of 0.0, justifying classification of the isolate tested to be of a low pathogenic phenotype [24]. A few chickens infected with SH2 shed virus though cloaca and seroconverted with low titer, which suggested the wild bird originated H11N9 viruses have not adapted to replicate in chicken [37]. After infection of mice with H11N9 virus at 10^3^ EID50 dose, the virus was able to replicate in lung without prior adaptation, though the replication of the virus was limited. And virus was also detected in other organs including nasal turbinate, brain, and colon. This was the first detection of H11N9 virus in nervous and digestive system of mice [16, 17, 20, 38]. After infection, no mice showed overt clinical signs; the inoculated mice showed slight weight loss at 4 and 10 days after infection before they started to regain weight.

In addition, full-genome sequenced progeny viruses from duck-derived SH2 H11N9 isolate included a series of known pathogenicity markers that play a role in determining high pathogenicity (Table 1). The substitution L89V in PB2, carried by the SH2 strain, is located in the region involved in interactions with PB1 (residues 51–259) and the region binding to the heat shock protein 90 (HSP90) (residues 1–515) [39]. G309D, T339K, R477G, and I495V are also located in the HSP90-binding region. These substitutions increase polymerase activity by enhancing the interaction of polymerase subunits or between polymerase and host factors to increase virulence in mammals [29, 40]. Additionally, most avian viruses, including SH2, have the PA protein residues R57, I62, and S65, which are located in a flexible loop (51–74) and play a major role in turning off host protein synthesis [30]. Surprisingly, the cleavage and polyadenylation specificity factor (CPSF30) binding site in the SH2 NS1 protein contains residues F103 and M106, which are common in human isolates, that stabilize CPSF30 binding and inhibit the production of beta interferon (IFN-β) mRNA [36]. Despite the lower significance of H11N9 LPAIVs for public health, antibody against influenza A/H11N9 has been reported in waterfowl hunters [18]. Significantly, H11N9 may donate NA gene to generate H7N9 virus, which mutated to be high pathogenic [14]. Thus, some reassortmentH11N9 viruses distributed in wild birds may possess new characteristics that have, having implications for public health, and requiring more extensive surveillance of avian reservoirs.

Furthermore, we analyzed the origin of the SH2 H11N9 virus. The HA and NA genes originated from A/duck/Jiangxi/22620/2012 (H11N9). Though A/wild bird/Anhui Shengjin Lake/S119/2014 (H11N9), A/wild bird/Anhui Caizi Lake/L306/2014 (H11N9), and A/waterfowl/Korea/S353/2016 [19] had similar genes, their phylogenetic relationships did not clearly indicate whether SH2 had obtained genes from them (Fig. 2; Fig. S1D; Fig. S1F). Apart from the NS gene, the other seven genes were similar to those of viruses in Japan and Korea in 2016. We assumed that viruses isolated in China in 2012–2015 had been transmitted to Japan and Korea by wild birds, and circulated locally in China at the same time. The H11N9 viruses in China, Japan, and Korea in 2016 may have been generated from independent reassortment events involving different NS genes. Rigorous wildlife disease surveillance was required to track the evolution of avian influenza virus, thus helping to reduce outbreak risk.

## Conclusions

In summary, we isolated a novel H11N9 strain in late 2016 that possesses new characteristics of increasing virulence. Although our phylogenetic analysis was based on strains with limited genetic diversity, SH2 is most likely a reassortant virus that originated from domestic birds in China. So, continued influenza surveillance in wild birds is extremely essential to prevent a potent pandemic caused by virus reassortment.

## Supplementary Information

Below is the link to the electronic supplementary material.

Supplementary file1 (PDF 1821 kb)

## Data Availability

GenBank accession numbers MW575006–MW575013. The datasets generated during and/or analysed during the current study are not publicly available due these records have not yet been released, but are available from the corresponding author on reasonable request. After released, the datasets generated during and/or analyzed during the current study would be available in the NCBI repository, https://www.ncbi.nlm.nih.gov/nucleotide.
