# Supplementary material for: Characterization of a reassortant H11N9 subtype avian influenza virus isolated from spot-billed duck in China
Source: Virus Genes. 2023 Jun 2;59(4):604–12. doi: 10.1007/s11262-023-02009-8 (PMC10235845; doi:10.1007/s11262-023-02009-8)
Supplement: Supplementary file 1 — Supplementary file1 (PDF 1821 kb) [file 11262_2023_2009_MOESM1_ESM.pdf]

Supplementary Material

Characterization of A Reassortant H11N9 Subtype Avian Influenza Virus Isolated from Spot-billed Duck in China

Bo Wang[1,2], Yanyi Huang[1,2], Bin Hu[1,2], Heng Zhang[1,2], Shuyi Han[1,2], Ziwen Yang[1,2], Qianqian Su[1], Hongxuan He[1]*

1 National Research Center for Wildlife-Borne Diseases, Institute of Zoology, Chinese Academy of Sciences, Beijing, China
2 College of Life Science, University of Chinese Academy of Sciences, Beijing, China

*** Correspondence:**
Corresponding Author: Hongxuan He, Ph.D., Executive Deputy Director of National Research Centre for Wildlife Borne Diseases, Institute of Zoology, Chinese Academy of Sciences. Add: No.1-5 Beichenxilu, Chaoyang District, Beijing 100101, P.R. China. Tel: 86-10-64807118(O/F).
E-mail: hehx@ioz.ac.cn

Supplementary Table 1. Primer sequences used in the study

Primer	Sequence (5'-3')
uni12	AGCRAAAGCAGG
MBTuni12	ACGCGTGATCAGCAAAAGCAGG
MBTuni13	ACGCGTGATCAGTAGAAACAAGG
Ba-PB2-1	TATTGGTCTCAGGGAGCGAAAGCAGGTC
Ba-PB2-2341R	ATATGGTCTCGTATTAGTAGAAACAAGGTCGTTT
Bm-PB1-1	TATTCGTCTCAGGGAGCGAAAGCAGGCA
Ba-PB1-2341R	ATATCGTCTCGTATTAGTAGAAACAAGGCATTT
Bm-PA-1	TATTCGTCTCAGGGAGCGAAAGCAGGTAC
Bm-PA-2233R	ATATCGTCTCGTATTAGTAGAAACAAGGTACTT

Supplementary Table 2. Dataset size and best-fit model for each gene segment

Gene	Taxa	Method	Substitution model‡	Clock model§	Tree model¶	Chain length/million
B2	143	MCC	GTR+I+G	uced	GMRF	100
PB1	139	MCC	GTR+I+G	uced	BSG	100
PA	138	MCC	GTR+I+G	uced	GMRF	100
HA	127	MCC	GTR+I+G	uced	BSG	100
NP	128	MCC	HKY+I+G	uced	BSG	100
NA	109	MCC	GTR+I+G	uced	GMRF	100
M	69	MCC	GTR+I+G	uced	EG	200
NS	120	MCC	HKY+I+G	uced	GMRF	100

† ML: Maximum Likelihood, MCC: Maximum clade credibility

‡ GTR+G: the General Time-Reversible substitution model with a gamma-distributed rate variation among sites, GTR+I+G: the General Time-Reversible substitution model with a proportion of invariant sites and gamma distributed rate heterogeneity

§ uced: uncorrelated exponential clock model

¶ BSG: Bayesian Skygrid, GMRF: GMRF Bayesian Skygrid, EG: Exponential Growth

Supplementary table 3. Homology of the genes of SH2 with related sequences

Gene	Virus name	Identify
PB2	A/mallard/Korea/H50-4/2016 (H5N3)	99.70%
PB1	A/mallard/Shanghai/NH011204/2018(H12N5)	99.52%
PB1	A/goose/Wuxi/7276/2016 (H3N8)	99.10%
PA	A/wild goose/dongting lake/121/2018(H6N2)	99.23%
PA	A/waterfowl/Korea/S017/2016 (H7N7)	98.80%
HA	A/duck/Ibaraki/F99/2016 (H11N9)	99.50%
NP	A/duck/Fukuoka/401202/2016 (H4N6)	99.94%
NA	A/duck/Ibaraki/99/2016 (H11N9)	99.72%
M	A/duck/Fukuoka/401202/2016 (H4N6)	99.90%
NS	A/wild bird/Jiangxi/P419/2016 (H6N8)	99.66%

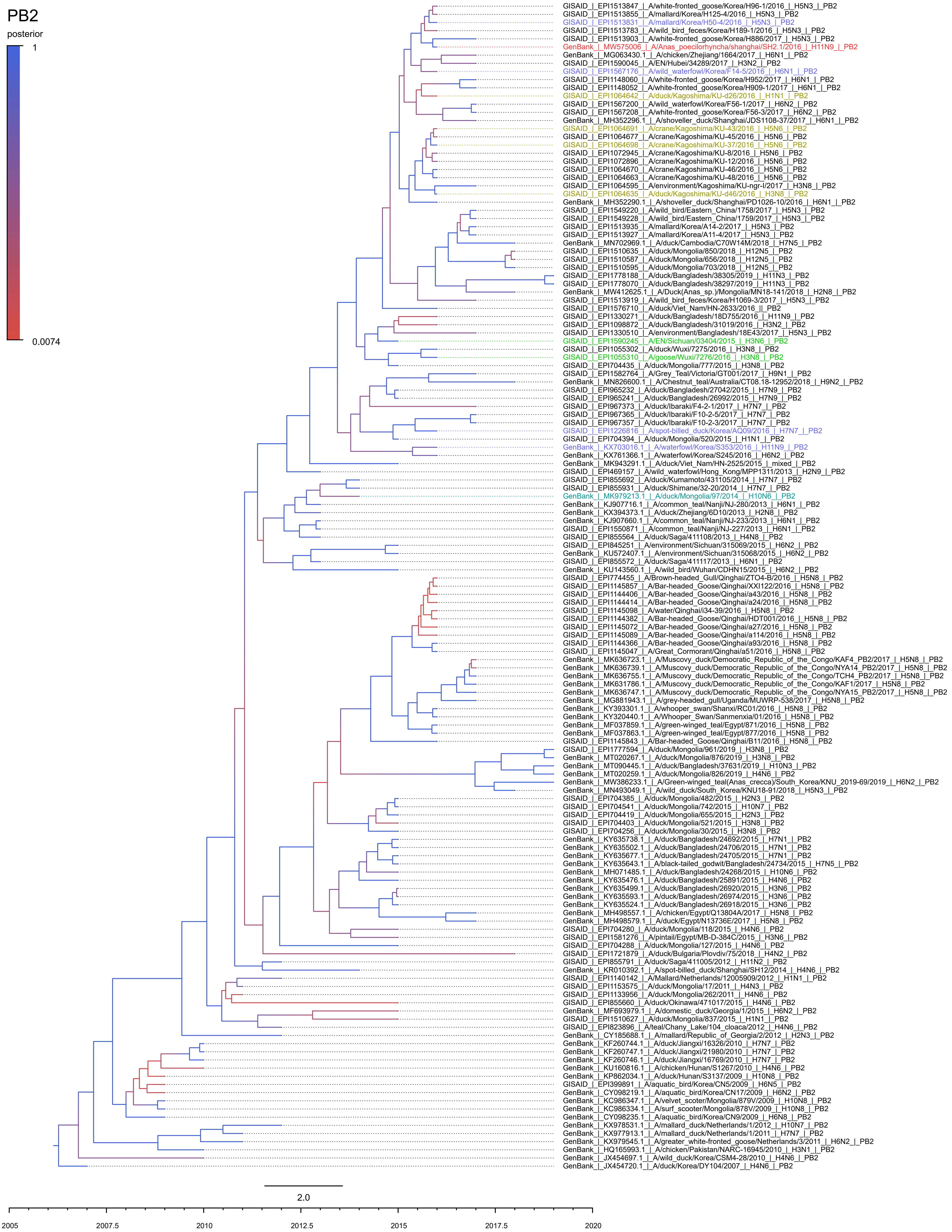

Fig S1 Genesis analysis of SH1 PB2 gene. The phylogenetic trees were conducted using gene sequences of top blast hits for SH2 in the NCBI and GISAID. SH1 is marked in red, and other related viruses in China, Japan, Korea and Mongolia are marked in green, yellow, purple and blue correspondingly. The tree was built by BEAST (v 1.8.4) and displayed using FigTree (v 1.4.2).

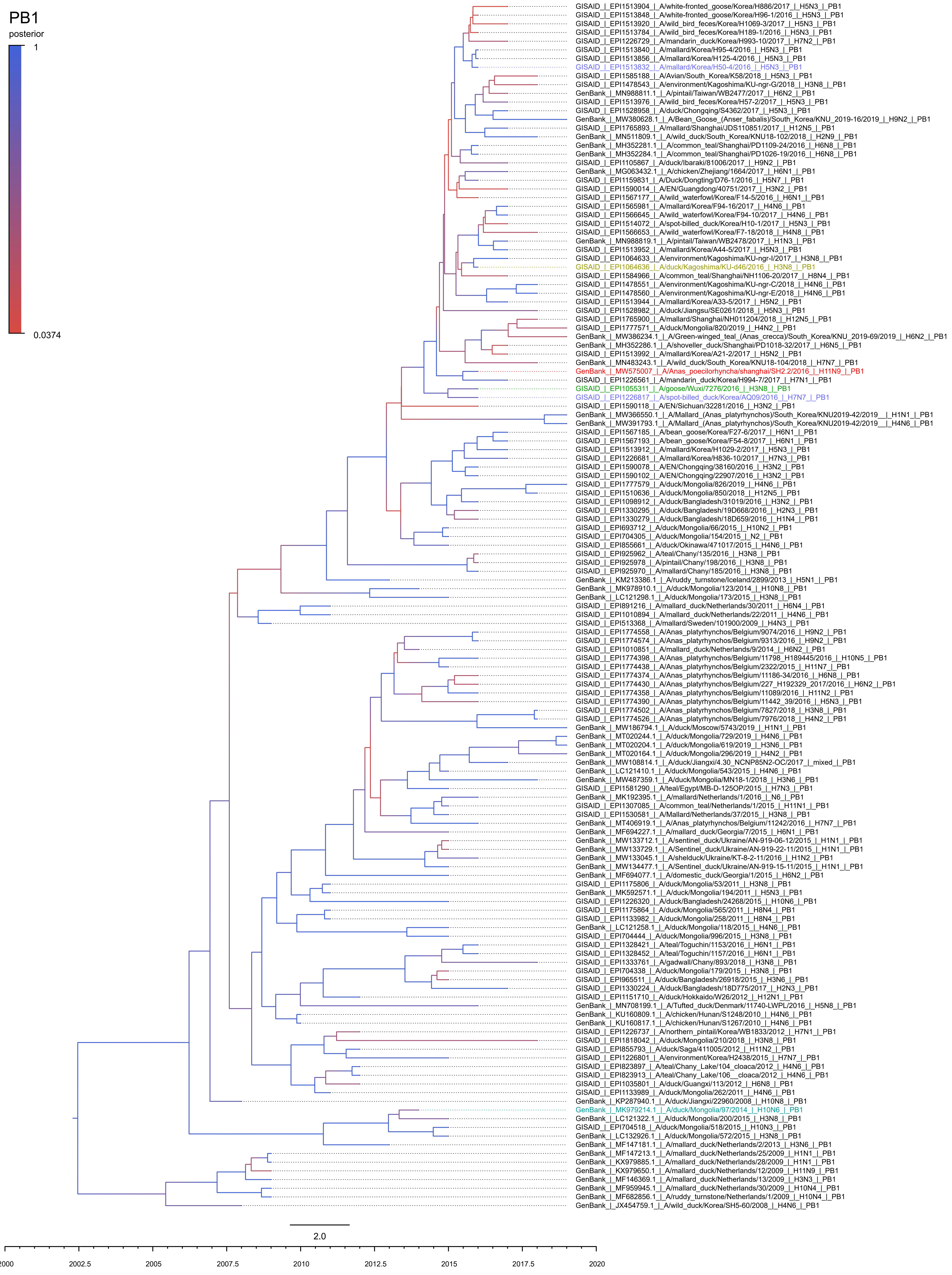

Fig S2 Genesis analysis of SH1 PB1 gene similar with Fig S1.

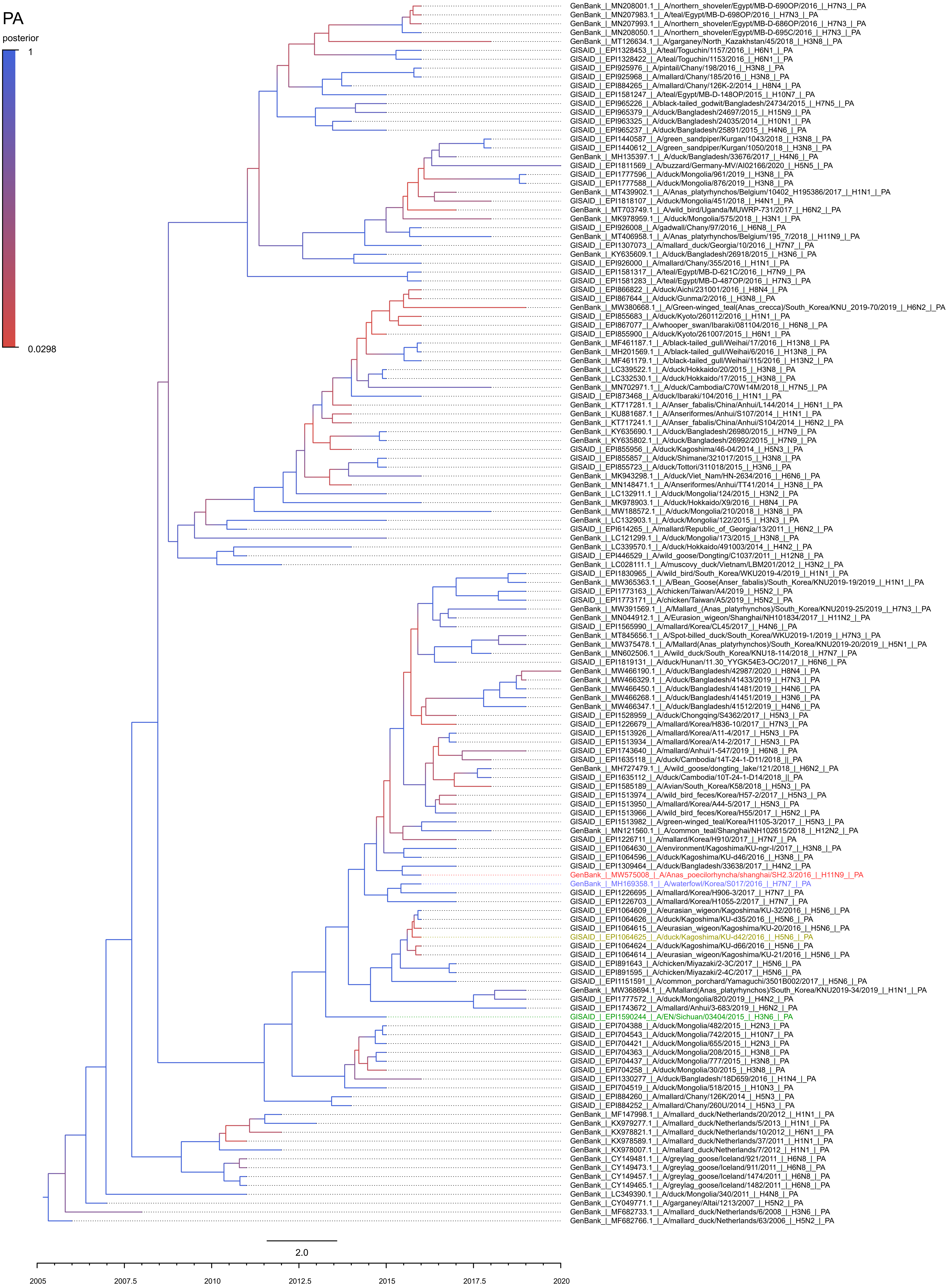

Fig S3 Genesis analysis of SH1 PA gene similar with Fig S1.

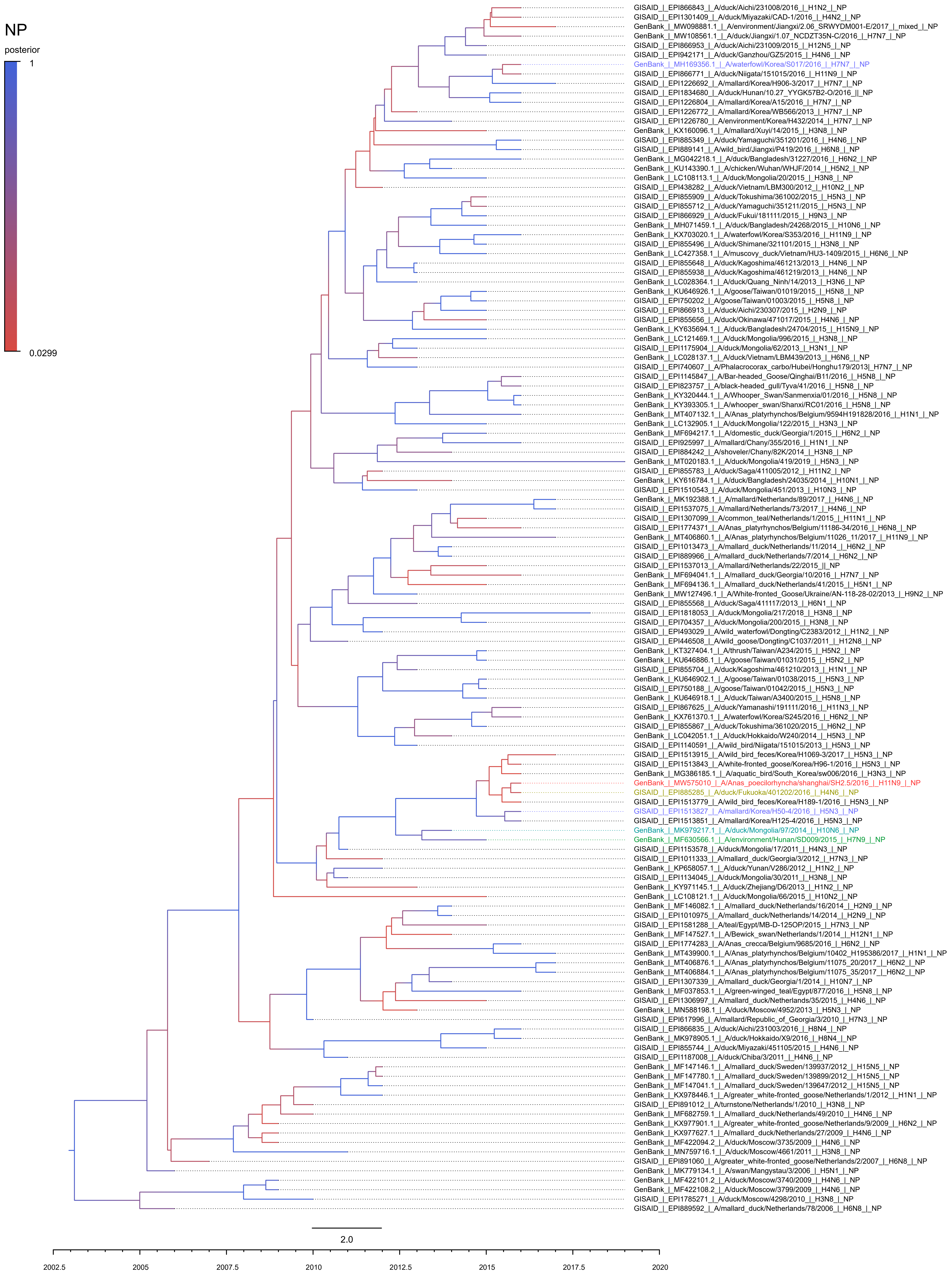

Fig S4 Genesis analysis of SH1 NP gene similar with Fig S1.

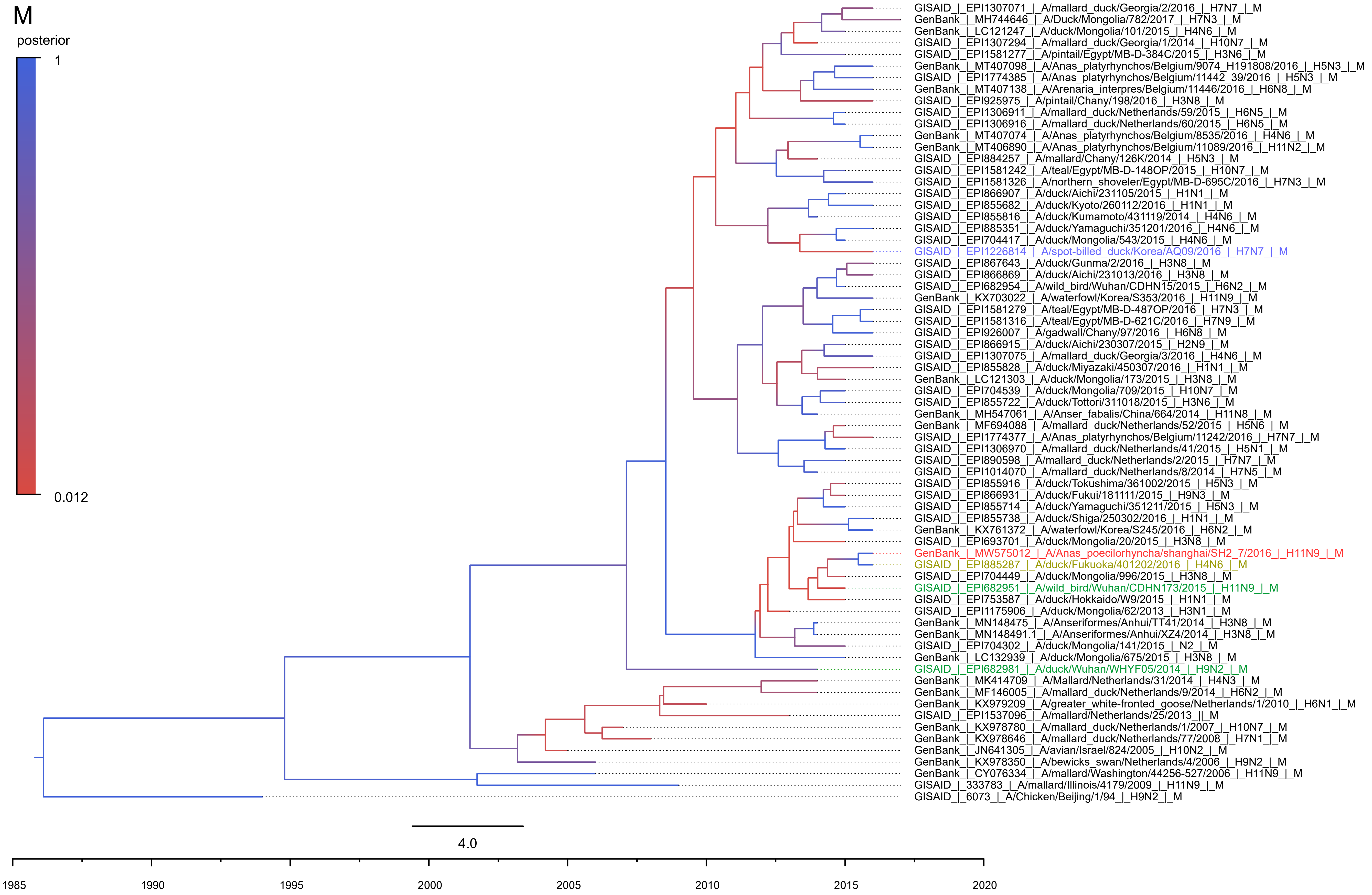

Fig S5 Genesis analysis of SH1 M gene similar with Fig S1.

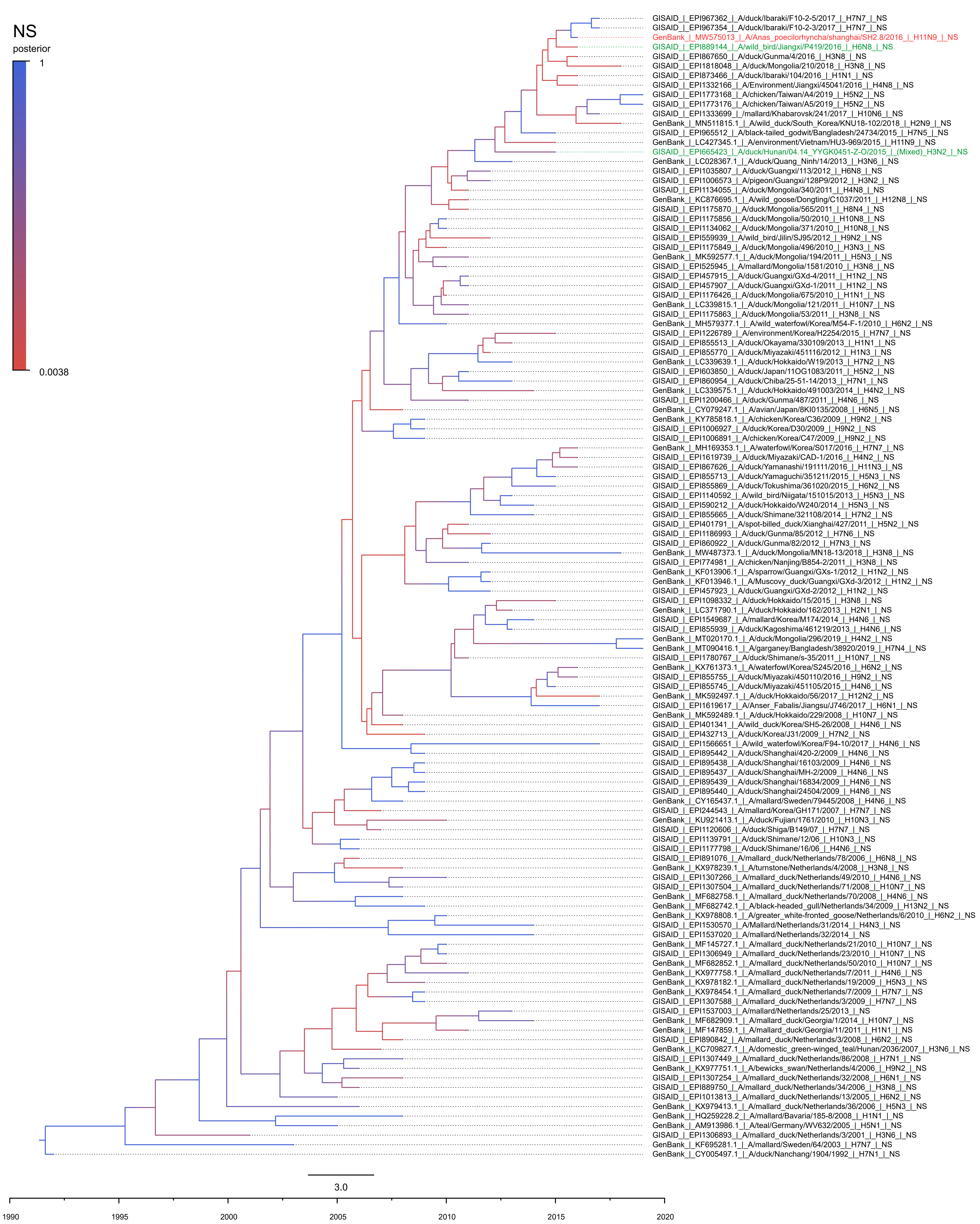

Fig S6 Genesis analysis of SH1 NS gene similar with Fig S1.
